# Application of cement-augmented pedicle screws in elderly patients with spinal tuberculosis and severe osteoporosis: a preliminary study

**DOI:** 10.1186/s13018-023-04099-4

**Published:** 2023-08-26

**Authors:** Shutao Gao, Yukun Hu, Fulati Mamat, Weidong Liang, Mardan Mamat, Chuanhui Xun, Jian Zhang, Weibin Sheng

**Affiliations:** Department of Spine Surgery, The First Affiliated Hospital of Xinjiang Medical University, 137 Liyushan Avenue, Xinshi District, Ürümqi, 830054 Xinjiang China

**Keywords:** Cement-augmented pedicle screws, Spinal tuberculosis, Osteoporosis, Polymethylmethacrylate

## Abstract

**Objective:**

Surgical management of elderly patients with spinal tuberculosis and severe osteoporosis is challenging. Cement-augmented pedicle screws (CAPS) have been specifically designed for elderly patients with osteoporotic spines. Herein, we investigated the feasibility of CAPS applied in elderly patients with spinal tuberculosis and severe osteoporosis.

**Methods:**

We retrospectively analyzed data of patients with spinal tuberculosis and severe osteoporosis between January 2017 and January 2021. Surgical data, including surgical duration and intraoperative blood loss, were recorded. Radiological parameters, such as correction of regional kyphotic angle and screw loosening, were also evaluated. Additionally, visual analog scores (VAS) and Oswestry disability index (ODI) were used to evaluate back pain and functional recovery, respectively. Erythrocyte sedimentation (ESR) and C-reactive protein (CRP) concentrations were detected to assess tuberculosis activity. The presence of complications and fusion rate was also assessed.

**Results:**

A total of 15 patients were included in this study. The surgical duration was 263.0 ± 56.2 min, with an average blood loss of 378.7 ± 237.0 ml. The correction of regional kyphotic angle was 12.4° ± 15.0°, and it was well maintained until the final follow-up. The mean VAS decreased from 6.0 ± 1.2 points to 0.5 ± 0.6 points, and ODI reduced from 37.8% ± 7.6% to 8.3% ± 2.8% (*P* < 0.01). At the final follow-up, ESR and CRP levels were within normal range. Bony fusion occurred in all patients, with an average fusion duration of 8.8 ± 1.5 months. No cases of pedicle screw pullout, screw loosening, or pseudoarthrosis occurred. Tuberculosis recurrence and dissemination were not observed during the follow-ups.

**Conclusions:**

CAPS fixation is an effective and safe technique to achieve solid fixation and favorable clinical outcomes in elderly patients with spinal tuberculosis and severe osteoporosis.

## Introduction

Spinal tuberculosis (TB) is the most common osteoarticular TB, amounting to approximately 50% of all cases [1]. The exact incidence and prevalence of spinal TB remain unclear; however, its incidence has been steadily increasing as poverty, population aging, and global migration increase [2–4]. Delays in timely diagnosis and initiation of proper treatment can lead to progressive kyphotic deformity, neurologic deficit, and disability [5].

Elderly individuals comprise a special population and often have poor general conditions and comorbidities; thus, they are more susceptible to spinal TB than young individuals. Wang et al. [6] reported that among 597 patients with spinal tuberculosis, 21.1% of the patients were aged over 60 years old. Spinal TB treatments include conservative therapy and surgery [1, 7]. Conservative therapies with anti-tuberculosis chemotherapy, bed rest, and nutritional support are the mainstay treatments for early-stage spinal TB [4]. However, due to the slow and insidious onset of this disease, it always presents in advanced stages [7]. For patients with progressive deformity, neurologic deficits, and large abscesses, surgical intervention may become inevitable [1, 8]. The main purpose of surgical treatment is to remove lesions, restore spinal stability, improve neurological function, and enable early activity [1].

Pedicle screws have excellent biomechanical properties and are the gold standard for rigid fixation in various spinal diseases. The robustness of traditional pedicle screws largely depends on the quality of the cancellous bone and the strength of the bone–screw interface [9]. Poor fixation strength may cause screw loosening and implant failure. Osteoporosis is reportedly a crucial risk factor for screw loosening and instrumentation failure [10, 11]. Therefore, in elderly patients with severe osteoporosis, instrumentation with traditional pedicle screws is challenging [12, 13].

Recently, expandable screws, curved screws, cortical bone trajectory screws, and cement-augmented pedicle screws (CAPS) have been introduced with improved pullout strength and a low risk of screw loosening [12]. By dosing cement through the screws into vertebral bodies, CAPS have been extensively used in patients with osteoporotic spines to increase the pullout strength [14]. However, vertebral infection post-cement augmentation is a life-threatening complication [15]. Reports have indicated that vertebral augmentation by cement may reactivate quiescent tuberculous lesions in elderly patients [16]. Li et al. [17] reported the application of polymethylmethacrylate (PMMA)-augmented screw fixation in elderly patients with thoracolumbar tuberculosis, while Li et al.’s study only included nine patients that underwent fixation with CAPS. Therefore, the clinical safety of CAPS applied in patients with spinal TB still needs further investigation. The objective of this study was to evaluate the feasibility of CAPS insertion in elderly patients with spinal TB and severe osteoporosis.

## Materials and methods

### Study population

Between January 2017 and January 2021, patients with spinal TB and severe osteoporosis who had undergone surgical treatment were retrospectively analyzed. Written informed consent was provided by all patients, and the protocol was approved by the ethics committee of our hospital. The diagnosis of spinal TB was based on medical history, clinical manifestations, and radiographic and laboratory examinations. Besides, pathological examinations were performed postoperatively to confirm the diagnosis. The bone mineral density (BMD) was estimated by dual-energy X-ray absorptiometry examination.

The inclusion criteria were: (1) a *T* score of BMD that was less than − 2.5 SD; (2) patients aged over 65 years; (3) patients with severe and/or progressive neurological impairment; (4) patients with poor response to anti-tuberculosis medications; and (5) a follow-up duration of more than 24 months. Correspondingly, the exclusion criteria were: (1) the presence of spinal diseases, such as congenital deformity, pyogenic spondylitis, ankylosing spondylitis, and parathyroid gland hyperfunction; (2) patients who were allergic to PMMA; and (3) patients with insufficient medical data.

### Preoperative management

X-ray, CT, and MRI were routinely performed to evaluate the extent of the lesions. All patients were regularly treated with anti-tuberculosis drugs of isoniazid, rifampicin, ethambutol, and pyrazinamide (HREZ) for 2–4 weeks preoperatively. For patients with severe malnutrition, anemia, and hypoproteinemia, nutritional support was recommended to improve their nutritional condition. Patients were scheduled for surgery when the poisoning symptoms significantly improved.

### Operative procedure

#### Posterior-only approach

Under general anesthesia, the patients were put in a prone position. A posterior midline incision was made to expose the affected vertebrae. Fenestrated pedicle screws were implanted at one or two levels above and below the affected vertebrae. PMMA bone cement was slowly injected under fluoroscopic guidance.

Unilateral or bilateral facetectomy followed by partial laminectomy was performed. Curettes were used to scrape off caseous necrosis, intervertebral disk, sequestrum, and granulated tissues. Abscess was drained by incubating a blunt-pointed negative pressure aspirator into the pus cavity. Subsequently, correction of kyphotic deformity was achieved by installing contoured rods with decompression maneuvers. Thereafter, 2.0 g streptomycin powder was topically applied. Allogeneic iliac bones were trimmed and embedded into the vertebral interbody. The rods were compressed to tighten the grafted bone and complete the kyphosis correction. A drainage tube was placed, and the incision was sutured in layers.

#### Combined anterior and posterior approach

The patient was placed in a lateral position. A 10-cm incision was made to expose the affected vertebrae through a retroperitoneal approach. Curettage of sequestrum, granulation tissue, and pus was done. Allogeneic iliac bones were installed into the interbody. Then, 2.0 g of streptomycin powder was topically applied to the lesion, and the incision was sutured in layers. Thereafter, the patient was placed in a prone position for posterior fixation with CAPS.

### Postoperative management and follow-up

Chemotherapy with HREZ was continued for 12–15 months postoperatively. All patients were prescribed calcium carbonate and vitamin D to improve bone mass [18–22]. Routine blood examination, hepatic and renal function examination, and erythrocyte sedimentation rate (ESR) and c-reactive protein (CRP) levels assessments were performed weekly during hospitalization and monthly after discharge. If adverse drug reactions such as abnormal liver and renal function occurred, the drug dosage was adjusted. All patients were examined clinically and radiologically at 1 week and 1, 3, 6, and 12 months postoperatively. Subsequent follow-ups occurred at one-year intervals.

### Observational parameters

The surgical data, including surgical duration, intraoperative blood loss, and surgery-related complications, were recorded. Visual analog scores (VAS) [23], Oswestry disability index (ODI) [24], and Frankel grade [25] were used to evaluate back pain and functional recovery. The regional kyphotic angle was measured to assess deformity correction [26]. ESR and CRP were assessed to monitor TB activity. The bony fusion was appraised by the presence of trabecular bone bridging between the vertebrae and the grafted bones. Pedicle screw pullout, screw loosening, tuberculosis dissemination, and tuberculosis recurrence were recorded to evaluate clinical effectiveness.

### Statistical analysis

Statistical analysis was performed using SPSS 22.0 software. Data of VAS, ODI, ESR, CRP, and regional kyphotic angle were presented as mean ± SD at different time points. Pre- and postoperative measurement data were compared using a paired t test. A *P* value less than 0.05 was considered statistically significant.

## Results

### Basic characteristics of the eligible patients

Totally, 15 patients (3 males and 12 females) were analyzed; the average age of all patients was 72.4 ± 6.4 years (range 65–85 years). Four patients had thoracic tuberculosis, one patient had thoracolumbar tuberculosis, and ten patients had lumbar tuberculosis. The average BMD was − 3.1 ± − 0.5 SD (range − 2.5 to − 4.0 SD). The detailed demographics of the included patients are displayed in Table 1.

**Table 1:** Clinical characteristics of the included patients

Patient ID	Age (yrs)/sex	Chief complaints	Affected segments	BMD (*T*-scores)	Comorbidity
1	81/F	Low back pain, radiating pain	L3-L4	− 3.8	Hypertension, pulmonary TB, anemia
2	72/F	Low back pain, radiating pain	L4-L5	− 2.7	Diabetes, chronic gastritis, anemia, malnutrition
3	78/F	Back pain, lower extremities weakness	T8-T9	− 2.9	Hypertension
4	69/F	Low back pain, radiating pain, lower extremities weakness	L1-L3	− 2.6	Cerebral infarction, pneumonia
5	71/M	Back pain	T9-T10	− 2.8	HIV, pulmonary TB, hepatitis B, anemia, malnutrition
6	69/F	Back pain, lower extremities weakness	T10-T11	− 2.7	Hypertension, coronary artery disease
7	83/F	Low back pain	L1-L3	− 3.2	Diabetes, hypertension, malnutrition
8	70/F	Low back pain, radiating pain	L3-L5	− 2.5	Diabetes, pulmonary TB
9	69/M	Low back pain, radiating pain	L3-L4	− 4.0	Pulmonary TB, hypertension, cardiac dysfunction
10	71/F	Low back pain, radiating pain	L2-L3	− 3.5	Hypertension
11	65/F	Low back pain, lower extremities weakness	T11-T12	− 3.2	Pulmonary TB, cardiac dysfunction
12	66/F	Low back pain, radiating pain	L4-L5	− 3.7	Hypertension, anemia, malnutrition
13	72/F	Radiating pain	L3-L4	− 2.9	Anemia, malnutrition
14	65/F	Low back pain, radiating pain	L4-L5	− 2.7	Cardiac dysfunction, anemia
15	85/M	Back pain	T12-L1	− 3.2	Pneumonia, lower limb thrombosis

*BMD* bone mineral density, *TB* tuberculosis, *AIDS* acquired immune deficiency syndrome

### Surgical approaches

Two patients underwent anterior debridement and bone grafting followed by posterior fixation. The other 13 patients underwent surgery via the posterior-only approach. The operation duration was 263.0 ± 56.2 min (range 160–350 min), and the estimated bleeding volume was 378.7 ± 237.0 ml (range 200–1100 ml). The detailed surgical data are displayed in Table 2.

**Table 2:** Details of treatment and outcome of the patients

Patient ID	Approach	Surgical duration (min)	Fixation segments	Blood loss (ml)	Cement leakage	ODI (%)		VAS		Frankel grade		Kyphotic angle (°)			Bony fusion (mons)	Follow-up (mons)
						Pre-Op	FU	Pre-Op	FU	Pre-Op	FU	Pre-Op	Post-Op	FU		
1	A-P	230	L2-L5	300	No	65	15	6	1	D	E	− 25	− 40	− 32	10	30
2	P	210	L3-S1	600	No	35	10	4	0	D	E	− 58	− 62	− 60	9	24
3	P	240	T5-T12	200	No	35	9	5	0	C	E	25	16	17	7	29
4	A-P	340	T10-L5	1100	No	30	6	7	0	C	E	49	6	10	12	53
5	P	260	T8-T11	200	No	40	8	5	0	E	E	15	10	11	8	41
6	P	340	T7-T11	500	No	40	10	6	0	D	E	7	5	8	9	26
7	P	160	T12-L4	250	No	47	15	8	2	C	E	1	4	4	8	40
8	P	300	L2-S1	300	No	35	11	5	0	D	E	− 40	− 41	− 45	10	35
9	P	230	L2-L5	300	No	35	8	6	0	D	E	− 41	− 48	− 50	7	36
10	P	225	T11-L5	400	No	60	20	5	0	E	E	27	− 13	− 18	8	24
11	P	350	T9-L2	500	No	45	10	8	1	C	E	20	− 12	− 9	7	32
12	P	220	L3-S1	200	No	40	6	7	1	D	E	− 39	− 50	− 45	11	50
13	P	280	L1-L4	250	Yes	45	10	6	1	D	E	− 28	− 44	− 42	8	25
14	P	320	L3-S1	400	No	50	10	7	1	D	E	− 22	− 34	− 30	8	29
15	P	240	T11-L3	180	No	55	12	5	1	D	E	-5	3	4	10	26

*Pre-Op* preoperative, *Post-Op* postoperative, *FU* latest follow-up, *P* posterior approach, *A* anterior approach, *A-P* combined anterior and posterior approach

### Clinical outcomes

The patients were followed-up for 33.3 ± 9.2 months (range 24–53 months). The mean ODI score decreased from 43.8% ± 10.1% (range 30–65%) preoperatively to 10.7% ± 3.7% (range 6–20%) at the final follow-up. The mean VAS score decreased from 6.0 ± 1.2 (range 4–8) preoperatively to 0.5 ± 0.6 (range 0–2) at the final follow-up. Four patients had Frankel grade C, nine had grade D, and two had grade C preoperatively. At the last follow-up, their neurological function significantly improved and was grade E (Table 2).

### Imaging findings

**Fig. 1:**
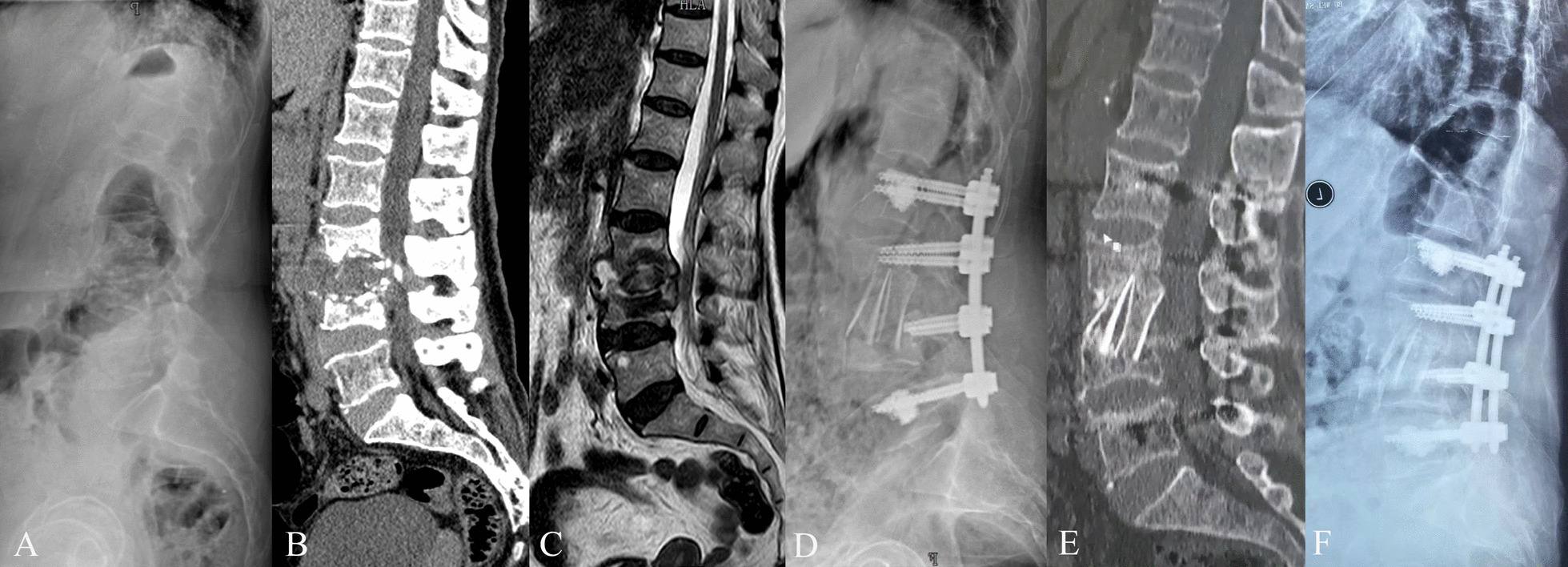
A 81-year-old female with L3-4 tuberculosis and severe osteoporosis. The patient underwent one-stage anterior debridement, bone grafting, and posterior percutaneous fixation with cement-augmented pedicle screws. **A**–**C** Preoperative lateral X-ray, sagittal CT scan, and sagittal MRI scan images demonstrated bone destruction and intraspinal abscess. **D** Postoperative lateral X-ray image. **E** Sagittal CT scan at 17-month follow-up. **F** Postoperative X-ray image at 30-month follow-up

**Fig. 2:**
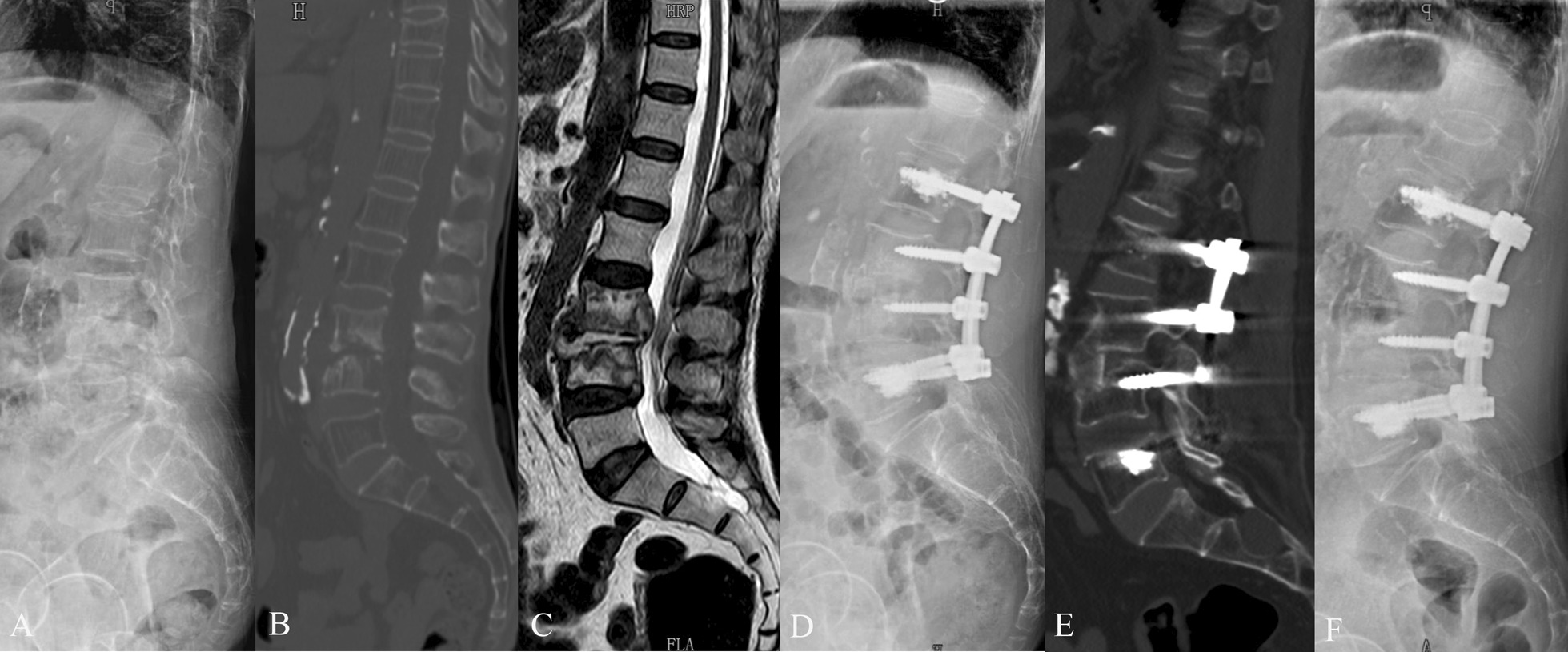
A 72-year-old female with L3-4 tuberculosis and severe osteoporosis. The patient underwent one stage posterior debridement, bone grafting, and fixation with cement-augmented pedicle screws. **A**–**C** Preoperative lateral X-ray, sagittal CT scan, and sagittal MRI scan images demonstrated bone destruction and intraspinal abscess. **D** Postoperative lateral X-ray image. **E** Sagittal CT scan at 8-month follow-up. **F** X-ray image at 25-month follow-up

**Fig. 3:**
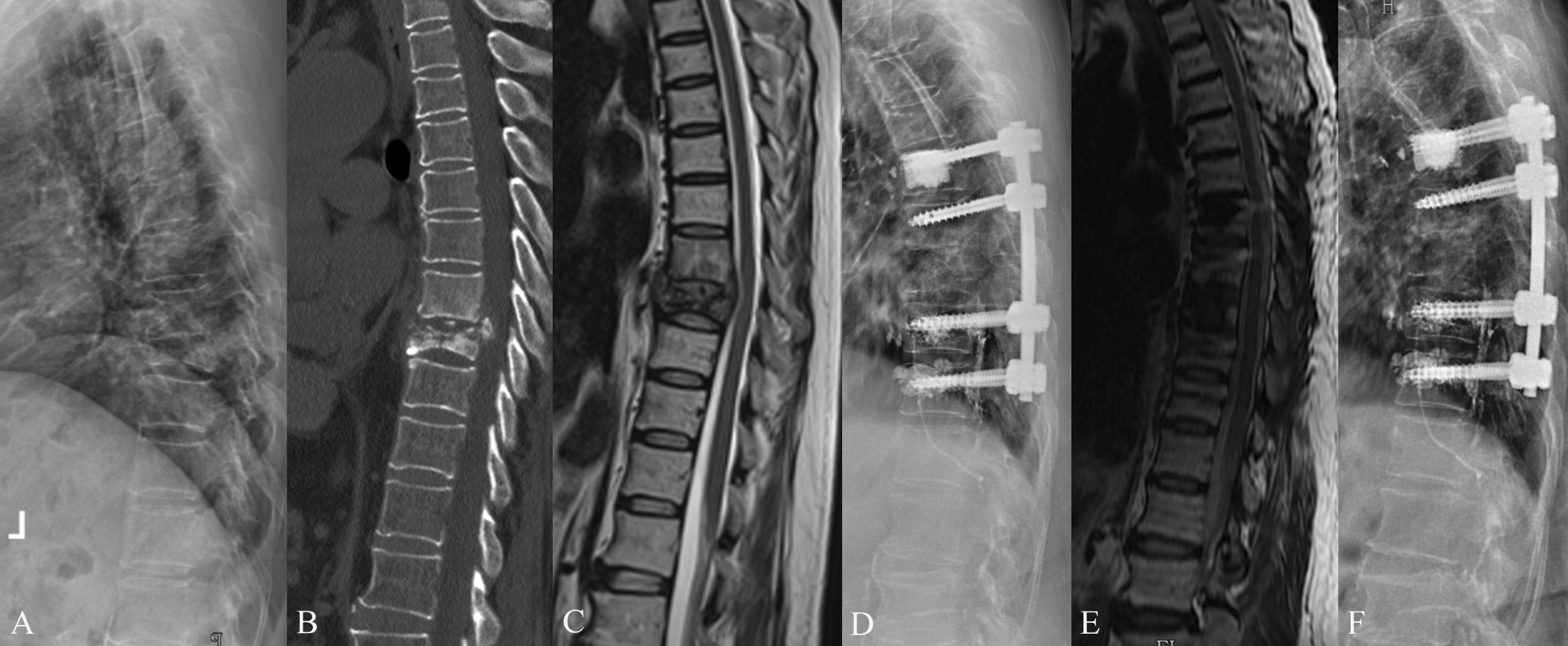
A 71-year-old male with T9-10 tuberculosis and severe osteoporosis. The patient underwent one stage posterior debridement, bone grafting, and fixation with cement-augmented pedicle screws. **A**–**C** Preoperative lateral X-ray, sagittal CT scan, and sagittal MRI scan images demonstrated bone destruction and sequestra. **D** Postoperative lateral X-ray image indicated cement leakage in the spinal canal and paravertebral vessel. **E** Sagittal MRI scan at 6-month follow-up. **F** X-ray image at final follow-up

Regarding the deformity correction, there was an improvement of 12.4° ± 15.0° in regional kyphotic angle postoperatively. The correction of regional kyphotic angle was 10.9° ± 15.2° at the final follow-up. Bony fusion was achieved in all patients, and the time to bony fusion was 8.8 ± 1.5 months (range 7–12 months) (Table 2). At the last follow-up, no cases of fixation failure or pseudoarthrosis occurred. The representative cases are displayed in Figs. 1, 2, and 3.

### Evaluation of TB activity

TB activity was evaluated by measuring inflammatory biomarkers, such as CRP and ESR. The preoperative CRP and ESR level were 31.5 ± 28.1 mg/L and 54.3 ± 13.3 mm/h, respectively. Three months postoperatively, the CRP and ESR levels reduced to 6.1 ± 2.1 mg/L and 19.9 ± 4.2 mm/h, respectively. At the last follow-up, the CRP and ESR levels were 4.3 ± 1.0 mg/L (*P* < 0.01) and 11.1 ± 2.4 mm/h (*P* < 0.01), respectively (Table 3).

**Table 3:** Control of tuberculosis infection

Patient ID	ESR (mm/h)					CRP (mg/L)					Tuberculosis dissemination	Recurrence
	Pre-Op	Post-Op	3-mons	6-mons	FU	Pre-Op	Post-Op	3-mons	6-mons	FU		
1	51	29	21	15	12	23	11	5	6	5	No	No
2	70	34	19	13	11	10	5	6	5	4	No	No
3	50	26	14	16	10	16	9	5	4	4	No	No
4	34	19	16	12	9	6	5	4	5	3	No	No
5	56	36	15	9	8	15	10	3	5	3	No	No
6	58	42	22	18	15	27	13	6	7	6	No	No
7	68	44	23	15	12	23	14	6	6	5	No	No
8	58	31	19	17	13	68	21	6	5	3	No	No
9	51	29	21	14	12	23	11	5	7	4	No	No
10	68	84	31	18	15	75	35	9	6	5	No	No
11	32	23	18	14	10	9	8	6	6	3	No	No
12	62	31	19	12	9	12	8	7	3	4	No	No
13	46	32	24	14	10	60	46	12	8	5	No	No
14	36	26	16	8	7	12	9	6	3	4	No	No
15	75	60	20	14	13	94	43	5	4	6	No	No

*ESR* Erythrocyte sedimentation, *CRP* C-reactive protein, *Pre-Op* preoperative, *Post-Op* postoperative, *FU* latest follow-up

### Complications

Cement leakage into the spinal canal and paravertebral vessel occurred in a patient while injecting the cement, and the cement was partially removed by laminar fenestration decompression (Fig. 3). The episode did not result in significant neurological deficit postoperatively. Cement leakage-induced pulmonary embolism were not seen in this case series. One patient developed pneumonia on postoperative day 5, and the patient was cured after administration of antibiotic therapy for 2 weeks. None of the patients developed dissemination or recurrence of spinal tuberculosis during the follow-ups.

## Discussion

Elderly patients with spinal tuberculosis and severe osteoporosis are usually bedridden for a long time and experience various complications [27]. Besides, such patients carry a higher risk of fracture and spinal cord damage [28]. Surgical treatment to remove the lesions and restore spinal stability may accelerate the recovery and enable early activity [7, 29, 30]. The stability and robustness of spinal fixation mainly depend on the purchase of the screws in the pedicle and vertebral body. Despite the wide use of pedicle screws in spine surgery to facilitate fusion and postoperative rehabilitation, osteoporosis-related issues including screw pullout, screw loosening, and fusion failure still limit their applicability [31]. BMD is a crucial factor that influences pedicle screws stability, and implant failures frequently occur in osteoporotic spines [32], which have brought attention to the design of the expandable pedicle screw, cortical bone trajectory screw, and CAPS to strengthen the purchase of the screws [12, 33].

In osteoporotic spines, PMMA cement augmentation is one of the most reliable techniques for achieving stability. CAPS have received considerable attention because they provide robust fixation and require a simple surgical procedure and have 1.5 times higher pullout force compared to traditional pedicle screws [34]. In a biomechanical study, Parè et al. [35] observed a significant increase in the pullout strength after injecting a small quantity of bone cement into osteoporotic vertebrae through a fenestrated pedicle screw. Amendola et al. [36] conducted a prospective study on 21 patients with poor bone quality who were treated with CAPS and found no screw loosening in the 81 inserted screws during a mean follow-up of 36 months. In the present case series, pedicle screw pullout and loosening were not observed intraoperatively and during follow-ups.

However, cement augmentation carries a risk of cement leakage [37], and cement leakage into the spinal canal may result in neurologic deficit, while leakage into vessels may cause pulmonary cement embolism (PCE) [38]. Following spinal cement augmentation, the incidences of symptomatic and asymptomatic PCEs are reportedly 1.2–1.4% and 4.2–16.3%, respectively [39]. PCE is significantly associated with the cement viscosity during its delivery. In this study, cement leakage into the spinal canal and paravertebral vessel occurred in a patient while injecting the cement, but no leakage-related neurologic deficits or PCE were observed postoperatively. The favorable outcomes could be ascribed to the fact that the PMMA cement was injected in its dough phase after examining its viscosity. Besides, continuous intraoperative fluoroscopy was carried out while injecting the PMMA cement to monitor leakage [40].

Dissemination of TB infection is another major concern during the use of CAPS. A new TB lesion may form by local reactivation of quiescent bacteria or by the release of mycobacteria from macrophages infected by TB bacilli that have migrated to the injury site [41, 42]. Besides, since bone cement is a foreign material, it carries a risk of infection. Park et al. [15] analyzed 826 patients with osteoporotic fractures that were treated with vertebroplasty or kyphoplasty and reported a 0.36% incidence of infection. Abdelrahman et al. [43] reported a 0.46% postoperative infection rate in a cohort of 1,307 patients who had undergone percutaneous vertebroplasty or kyphoplasty. Zou et al. [16] reported two patients in whom spinal tuberculosis occurred after vertebroplasty and kyphoplasty. Despite the low infection rate, postoperative infection always results in life-threatening complications in frail patients with notable comorbidities [43]. Therefore, some scholars avoid cement augmentation techniques in patients with preoperative infectious diseases [15, 43].

In this case series, no patients developed TB dissemination. To avoid hematogenous TB infection seeding, implanting of the pedicle screws and injection of PMMA were performed before decompression and debridement of the TB focus in the posterior-only approach surgery. Besides, the patients were prescribed anti-tuberculosis drugs for 2–4 weeks preoperatively. Patients were not scheduled for surgery until their poisoning symptoms and nutritional condition improved significantly. Healthy vertebrae were determined by MRI examination, and CAPS were inserted only in the vertebrae without abnormal signal changes.

The regional kyphotic angle, which is a crucial indicator for deformity correction, had significantly improved after surgery. All the patients had satisfactory outcomes for back pain and neurological function. Besides, there was no pedicle screw pullout, screw loosening, pseudoarthrosis, or tuberculosis recurrence in this study. The favorable outcomes occur due to complete debridement, robust bone grafting and fixation, and effective anti-tuberculous therapy.

For managing bone defects after debridement, an autologous iliac crest is the most favorable graft in adults. However, because the patients had accompanied severe osteoporosis, the autogenous iliac crest could not provide solid and reliable intervertebral support. Therefore, allogenous iliac bone was used as the grafting material in this case series. Bony fusion occurred in all cases, suggesting iliac bone allograft was a satisfactory alternative to autograft.

This study had some limitations should be pointed out. First, the nature of this retrospective study determined the level of evidence was low, and further prospective studies are encouraged. Second, this study was conducted in a single center with a small sample size, and the results might be biased. Multicenter studies with long-term follow-up data would help draw a more reliable conclusion.

## Conclusion

CAPS fixation is an effective and safe technique to achieve solid fixation and favorable clinical outcomes in elderly patients with spinal TB and severe osteoporosis.

## Data Availability

The datasets are available from the corresponding author upon reasonable request.

## Abbreviations

CAPS: Cement-augmented pedicle screws
TB: Tuberculosis
VAS: Visual analog scores
ODI: Oswestry disability index
ESR: Erythrocyte sedimentation
CRP: C-reactive protein
